# Continuous monitoring of left ventricular function in postoperative intensive care patients using artificial intelligence and transesophageal echocardiography

**DOI:** 10.1186/s40635-024-00640-9

**Published:** 2024-06-10

**Authors:** Jinyang Yu, Anders Austlid Taskén, Erik Andreas Rye Berg, Tomas Dybos Tannvik, Katrine Hordnes Slagsvold, Idar Kirkeby-Garstad, Bjørnar Grenne, Gabriel Kiss, Svend Aakhus

**Affiliations:** 1 Department of Circulation and Medical Imaging, Faculty of Medicine and Health Sciences, NTNU, Norwegian University of Science and Technology, Postboks 8905, 7491 Trondheim, Norway; 2 Clinic of Cardiology St. Olav’s Hospital, Trondheim University Hospital, Trondheim, Norway; 3 Department of Computer Science, Norwegian University of Science and Technology, Trondheim, Norway; 4 Department of Anesthesia and Intensive Care, St. Olav’s Hospital, Trondheim University Hospital, Trondheim, Norway; 5 Clinic of Cardiothoracic Surgery, St. Olav’s Hospital, Trondheim University Hospital, Trondheim, Norway; 6 Centre of Innovative Ultrasound Solutions, Trondheim, Norway

**Keywords:** Transesophageal echocardiography, Deep learning, Left ventricular function, Hemodynamic monitoring, Mitral annular plane systolic excursion, Critical care, AutoMAPSE

## Abstract

**Background:**

Continuous monitoring of mitral annular plane systolic excursion (MAPSE) using transesophageal echocardiography (TEE) may improve the evaluation of left ventricular (LV) function in postoperative intensive care patients. We aimed to assess the utility of continuous monitoring of LV function using TEE and artificial intelligence (autoMAPSE) in postoperative intensive care patients.

**Methods:**

In this prospective observational study, we monitored 50 postoperative intensive care patients for 120 min immediately after cardiac surgery. We recorded a set of two-chamber and four-chamber TEE images every five minutes. We defined *monitoring feasibility* as how often the same wall from the same patient could be reassessed, and categorized monitoring feasibility as *excellent* if the same LV wall could be reassessed in ≥ 90% of the total recordings. To compare autoMAPSE with manual measurements, we rapidly recorded three sets of repeated images to assess precision (least significant change), bias, and limits of agreement (LOA). To assess the ability to identify changes (trending ability), we compared changes in autoMAPSE with the changes in manual measurements in images obtained during the initiation of cardiopulmonary bypass as well as before and after surgery.

**Results:**

Monitoring feasibility was excellent in most patients (88%)*.* Compared with manual measurements, autoMAPSE was more precise (least significant change 2.2 vs 3.1 mm, *P* < 0.001), had low bias (0.4 mm), and acceptable agreement (LOA − 2.7 to 3.5 mm). AutoMAPSE had excellent trending ability, as its measurements changed in the same direction as manual measurements (concordance rate 96%).

**Conclusion:**

Continuous monitoring of LV function was feasible using autoMAPSE. Compared with manual measurements, autoMAPSE had excellent trending ability, low bias, acceptable agreement, and was more precise.

**Graphical Abstract:**

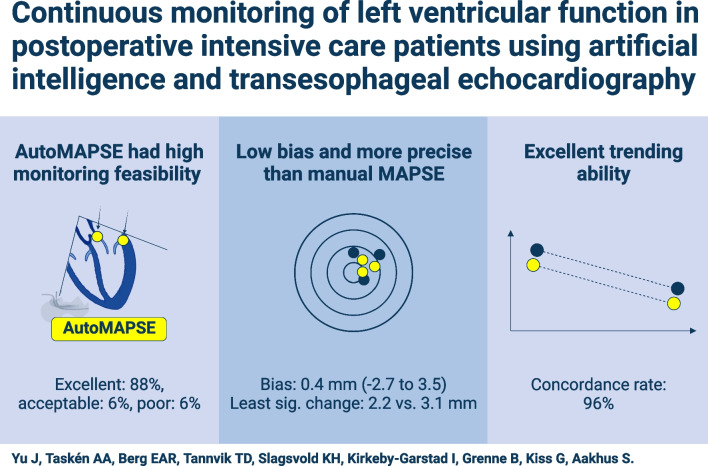

**Supplementary Information:**

The online version contains supplementary material available at 10.1186/s40635-024-00640-9.

## Introduction

Unexpected development of left ventricular (LV) dysfunction is common in intensive care patients [1, 2] and has implications for patient management [3–5] as well as the prognosis [6–9]. Monitoring is imperative to detect early deterioration of LV function. Unfortunately, appropriate assessment of LV function requires echocardiography, a method that is too time-consuming for continuous monitoring.

To address the need for continuous monitoring of LV function, we have developed a method that combines transesophageal echocardiography (TEE) with artificial intelligence (AI) to measure mitral annular plane systolic excursion (MAPSE) automatically (autoMAPSE) [10–12]. MAPSE reflects LV longitudinal function, is highly correlated with global longitudinal strain [13], and has superior prognostic ability compared to LV ejection fraction in intensive care patients [14]. AutoMAPSE offers two important advantages compared to previous solutions that combine AI and transthoracic echocardiography [15–18]. First, the TEE probe is relatively stable and allows for hands-free image acquisitions. Second, MAPSE relies less on image quality as compared to LV ejection fraction or global longitudinal strain. Thus, the combined use of hands-free TEE and AI to automatically measure MAPSE could potentially provide continuous monitoring of LV function.

In this study, we tested the hypothesis that hands-free autoMAPSE could provide continuous monitoring of LV function by determining its feasibility, precision, and agreement with manual measurements of MAPSE in postoperative intensive care patients.

## Methods

### Patients and ethics

This prospective observational study included 51 patients scheduled for on-pump cardiac surgery with an indication for perioperative TEE. They were included between October 2022 and March 2023 at St Olav’s University Hospital, Trondheim, Norway, and excluded if they were < 18 years old, unwilling to participate, or had clinical contraindications to TEE [19]; poor image quality or arrhythmias were not exclusion criteria. Due to study resources, we included a maximum of one patient daily. Our study did not interfere with standard clinical care. This study was performed in line with the principles of the Declaration of Helsinki. Approval was granted by the Regional Committee for Ethics in Medicine (REK number 2022/345556). All patients provided written informed consent prior to participating. We adhered to the Strengthening the Reporting of Observational Studies in Epidemiology (STROBE) checklist [20] when composing this manuscript.

### Study design

**Fig. 1:**
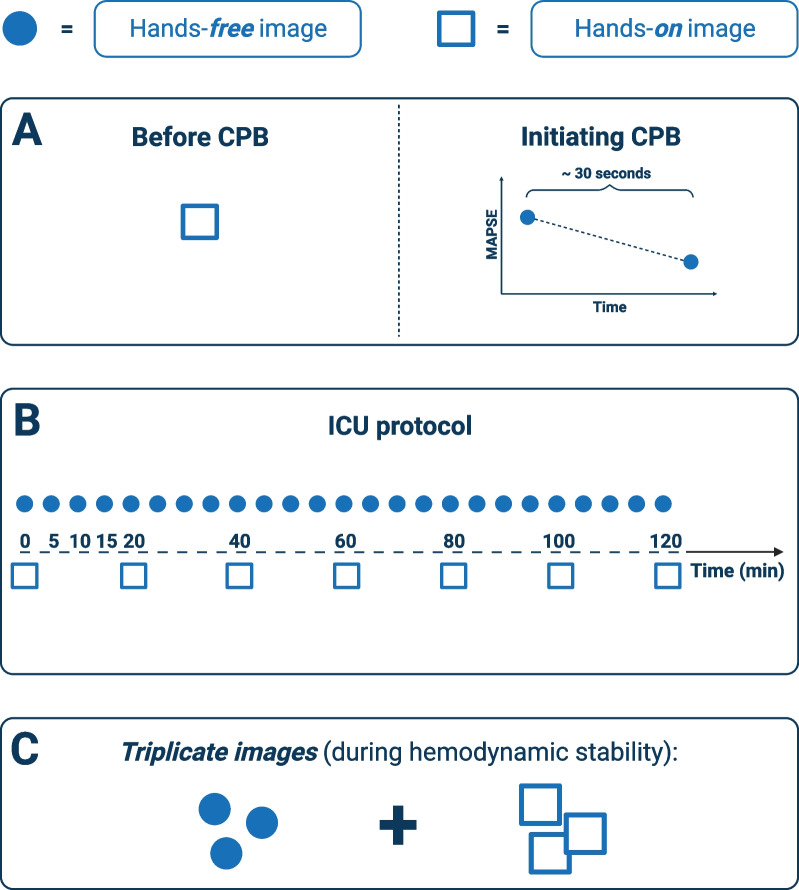
Study design. **A** One set of hands-on images was recorded before cardiopulmonary bypass (CPB) and two sets of hands-free images were recorded when initiating CPB. **B** One set of hands-free images was recorded every five minutes, and one set of hands-on images was recorded every 20 min. **C** Triplicate images were taken in rapid succession when the patients were hemodynamically stable in the intensive care unit (ICU). Filled circles, hands-free images; open squares, hands-on images. *CPB* cardiopulmonary bypass, *ICU* intensive care unit,  *MAPSE* mitral annular plane systolic excursion

To assess the various requirements for monitoring, we recorded several sets of hands-on and hands-free images in the operating room (Fig. 1A) as well as in the intensive care unit (ICU) immediately after surgery (Fig. 1B and C).

#### Image acquisition

Hands-on images were obtained by retroflecting and turning the probe with the omniplane at 90 degrees to acquire a mid-esophageal two-chamber (2C) view without foreshortening. Then, we used simultaneous biplane imaging (Multi-D®, GE, Healthcare, Horten, Norway) and aligned the secondary sector through the LV apex. Next, we rotated the omniplane angle in the secondary sector to approximately 0 to 30 degrees to acquire an optimized mid-esophageal four-chamber (4C) view. The reason for using biplane imaging from 2C, rather than 4C, was because visualization of the LV apex is often not possible in the 4C using TEE. Finally, we maximized the sector width and increased the temporal resolution to at least 40 frames per second.

The hands-free images were acquired by leaving the tip of the probe passive in the esophagus after acquiring the hands-on images. We used single-plane imaging to compensate for the reduced image quality. The temporal resolution was not adjusted. To allow prolonged hands-free imaging, we secured the probe with a custom-made probe holder (Supplementary Video 1). For consistency, the hands-free images were obtained with a maximized sector width and predefined omniplane rotations (90 degrees for 2C and 30 degrees for 4C) unless stated otherwise. The image was adjusted to include the mitral annulus from at least one wall. To minimize the risk of esophageal trauma, we ensured that the probe tip was always unlocked during prolonged hands-free imaging [21, 22].

Each set of images consisted of 2C and 4C views, providing MAPSE from up to four LV walls. To increase precision, we recorded up to ten heartbeats and measured MAPSE on all available beats. Thus, we reported one measurement of MAPSE as the average MAPSE of up to ten heartbeats for each wall at a specific time point. All images were recorded using a state-of-the-art scanner (E95, GE, Healthcare, Horten, Norway) with a 6VT-D probe (GE, Healthcare, Horten, Norway). A trained researcher with three years of clinical anesthesiology training (J.Y.) recorded all the images.

#### ICU protocol

To assess hands-free autoMAPSE for continuous monitoring, we monitored each patient for 120 min starting immediately after they arrived at the ICU (Fig. 1B). The TEE-probe remained in situ after surgery. Every five minutes, we recorded a set of hands-free images; each set comprised ten consecutive heartbeats of 2C and 4C views (Fig. 1B).

We also recorded a set of hands-on images every 20 min (Fig. 1B); each set comprised, again, ten consecutive heartbeats of simultaneous biplane images of 2C and 4C views. At each 20-min interval, we recorded the hands-free images before the hands-on images. Later, we compared the automatic measurements from the hands-on images with the preceding hands-free images to assess the effect of small probe adjustments. Outside each scheduled 20-min interval, we adjusted the probe only if the LV was completely displaced from the image. We noted these displacements, recorded a set of hands-free images, and adjusted the probe.

#### Definitions of monitoring feasibility

To continuously monitor LV function, MAPSE of the same wall for the same patient must be reassessed over time. To quantify reassessment over time, we first defined hands-free autoMAPSE as feasible if it measured MAPSE from at least one wall at a specific time point (Fig. 1B). We based this criterion on reports showing that MAPSE from any wall reflects global LV function, not the regional LV function of that wall [23–26]. Then, we defined *monitoring feasibility* as how often we could reassess the same wall from the same patient during the ICU protocol. If it was ≥ 90% of the total recordings, we categorized monitoring feasibility as *excellent*. If it was 50–90%, we categorized it as *acceptable*, and if it was < 50%, we categorized it as *poor.*

#### Triplicate images

To assess the precision of each method, i.e. hands-on and hands-free image recordings, we recorded a triplicate set of images in the ICU during a period of stable LV function (Fig. 1C). Within a time frame of less than one minute, we assumed stable LV function if the patient had otherwise stable mean arterial pressure, heart rate, and drug infusions. Within this short time frame, we rapidly recorded a triplicate set of hands-on and hands-free images.

#### Trending ability

To assess the ability of autoMAPSE to detect changes in manual measurements (trending ability), we recorded images at two different time points where we expected changes in MAPSE. Trending ability for hands-on and hands-free images was assessed differently. For the hands-on images, the first time point was recorded during the clinical echocardiography examination before cardiopulmonary bypass (CPB, Fig. 1A), and the second time point was during the recording of the triplicate images in the ICU (Fig. 1C).

For the trending ability of hands-free images, we used a prolonged recording (approximately 30 s) during the initiation of CPB (Fig. 1A). When initiating CPB, venous return is drained into the extracorporeal circuit, reducing LV preload and MAPSE within seconds. The acquisition of the hands-free images was slightly different during the initiation of CPB; in order to obtain MAPSE from all four walls, we used simultaneous biplane imaging with maximized sector size and temporal resolution of at least 40 frames per second. Like the other hands-free images, the probe’s position was stabilized externally, and the probe tip was kept passive. The first time-point was the average MAPSE of the first three-to-five heartbeats of this prolonged recording, while the second was the average MAPSE of three-to-five heartbeats after full CPB.

### Echocardiographic measurements

We analyzed the images using both autoMAPSE and manual measurements, resulting in four different echocardiographic measurement methods: (1) hands-free autoMAPSE, (2) hands-on autoMAPSE, (3) hands-free manual MAPSE, and (4) hands-on manual MAPSE.

#### Manual measurements of MAPSE

**Fig. 2:**
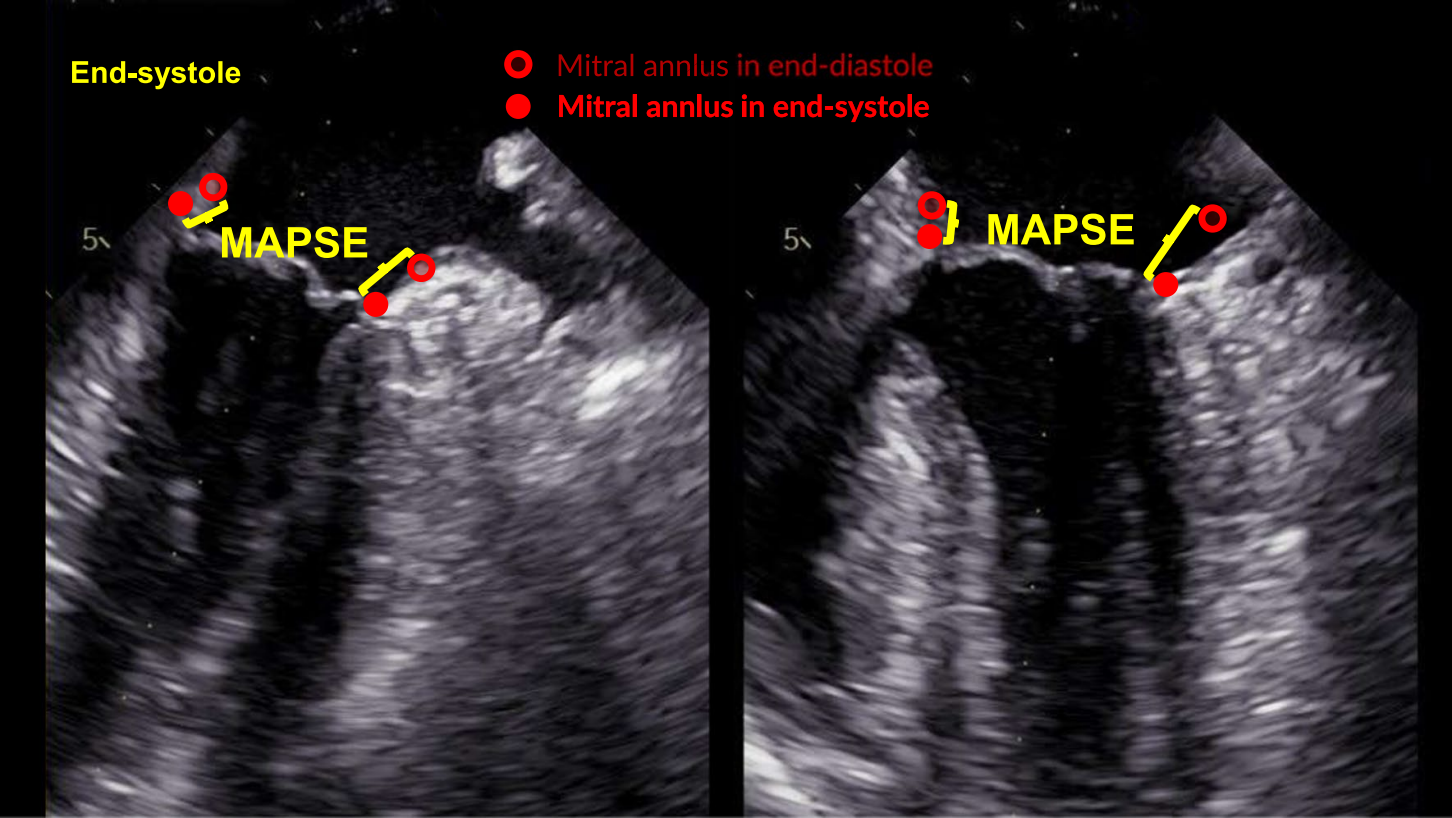
End-systolic image of mid-esophageal two- and four-chamber views to demonstrate the manual measurements of mitral annular plane systolic excursion (MAPSE) on B-mode images. For each of the four walls, the mitral annulus is identified in end-diastole (open circles) at its the highest position, and in end-systole (solid circles) at its lowest position. MAPSE is the distance between end-diastole and end-systole (yellow brackets). MAPSE, mitral annular plane systolic excursion

We manually measured the MAPSE of every wall using calipers directly on B-mode images in EchoPAC software (version 204, GE Healthcare, Horten, Norway). Again, we averaged the MAPSE from ten heartbeats for increased precision. Details regarding the technique for manual measurements of MAPSE using TEE (Fig. 2) have previously been presented by our group [10]. The interobserver variability of this manual method has been previously reported (intraclass correlation of 0.86, bias − 0.9 mm, limits of agreement [LOA] − 4.7 to 3.0 mm) [10]. We rated the image quality of the hands-free images as suboptimal if we could not clearly see the annulus in more than two of the four walls. The images from the ICU protocol were not manually measured (Fig. 1B).

#### Automatic measurement of MAPSE using artificial intelligence

**Fig. 3:**
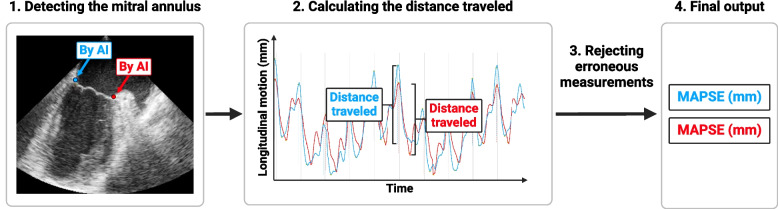
Overview of the pipeline of autoMAPSE. **1** For each frame, the mitral annulus is automatically detected using artificial intelligence, indicated by the red and blue dots. **2** For each cardiac cycle (vertical lines), MAPSE is the distance between the highest and the lowest point. The brackets demonstrate the MAPSE of one cardiac cycle. The longitudinal motion of the mitral annulus is measured for all cardiac cycles for the entire recording. **3** Erroneous measurements (none in the example image) are rejected automatically by the filtering algorithms described in the main text. **4** The final output is the average MAPSE of all cardiac cycles within each wall. *AI* artificial intelligence, *MAPSE* mitral annular plane systolic excursion

We used autoMAPSE on all the recorded images, obtaining the MAPSE of each of the four LV walls automatically. AutoMAPSE is a custom-made software we have developed, validated [10, 11], and refined [12] at our institution. The autoMAPSE pipeline comprises a convolutional neuronal network that was trained under supervised learning to detect the mitral annulus in TEE images [12], as well as a set of filtering algorithms for rejecting erroneous measurements (Fig. 3) [10]. The refined version of autoMAPSE used in this present study incorporates spatial and temporal information, which improves the consistency of predicting the frame-to-frame position of the mitral annulus [12]. After detecting the mitral annulus throughout each heartbeat, autoMAPSE measures the distance traveled by the mitral annulus from the highest to the lowest position within each wall and heartbeat [10]. The filtering algorithms rejected the autoMAPSE measurement if (1) the mitral annulus was detected more than 5 mm apart between two frames, (2) the mitral annulus was detected in less than 60% of the frames per heartbeat, and (3) the highest position of the mitral annulus was not detected around the R-wave of the electrocardiogram [10]. The final output is the MAPSE of the two LV walls for each recording, reported in millimeters after averaging all feasible heartbeats for each wall. Further technical details are found elsewhere [10, 12].

### Statistical analysis

To compare autoMAPSE with manual measurements, we used the outcomes bias, limits of agreement (LOA), the precision of each method, and trending ability. Bias, LOA, and precision were assessed using the triplicate images (Fig. 1C). Because each patient had triplicate measurements from four LV walls, the measurements had a within-patient dependency. Unless adjusted for, this within-patient dependency will cause the LOA to be underestimated [27, 28]. We made this adjustment using the linked replicates model described by Carstensen [28]. This approach modifies the Bland–Altman analysis using a linear mixed model with restricted maximum likelihood, where the fixed effects were *measurement method* and *patient*, and the random effects were *measurement method-patient interaction* and *patient-MAPSE interaction* [28]. Because the imprecision of manual measurements independently widens the LOA, the utility of autoMAPSE could be falsely rejected [29, 30]. When validating AI in echocardiography, a common solution to this issue is to target the interobserver variability [15–17]. Thus, we defined acceptable agreement a priori based on the previously established interobserver variability using the same manual measurement method for MAPSE as ours (LOA − 4.7 to 3.0 mm) [10].

To calculate the precision of each method, we used the residual standard deviation (SD) of each measurement method from the linked replicates model and reported the precision as the least significant change (LSC) using the formula [28, 31]:

$$LSC= \sqrt{2}\times 1.96 \times \frac{Standard\; deviation}{\sqrt{Number\; of\; measurements}}$$

In this formula, each *measurement* was the average MAPSE of ten heartbeats from one LV wall, not the MAPSE of one heartbeat.

We assessed the trending ability using a four-quadrant plot and reported the concordance rate with and without a central exclusion zone of 10% change (0.7 mm) [32]. Using an exclusion zone increases the signal-to-noise ratio, and we adapted the zone of 10% change from validation methods for cardiac output monitors [32]. We defined trending ability as excellent if concordance rates were ≥ 95%, and good if concordance rates were ≥ 80%. Finally, we assessed the significance of each method's change in MAPSE by fitting a linear mixed model for each method. In each model, *MAPSE* was the dependent variable, *time point* was the fixed factor, and *walls* and* patients* were random factors.

We reported central tendencies as mean (SD) if data were Normally distributed, otherwise as median [interquartile range]. The normality of distributions was assessed by visual inspection of histograms. The threshold for statistical significance was set at *P*-value < 0.05. Missing data were not replaced. The statistical analysis plan was established a priori. The sample size was decided based on our experience with similar method comparison studies. We used Stata 17.0 (StataCorp LLC) for all statistical analyses.

## Results

### Patient characteristics

We enrolled 51 patients after cardiac surgery. In one patient, images were obtained when initiating CPB, but the patient had to be excluded after ICU arrival due to a leaking endotracheal cuff. The leak was unrelated to the TEE probe. Thus, 50 patients were monitored in the ICU (Table 1) and no patients suffered complications from TEE. One patient was only monitored for 100 min due to a decision for early extubation, and a second patient was monitored for 40 min due to emergent reoperation. In a third patient, the data for trending ability were missing due to the investigator's absence. The median temporal resolution was 46.2 [40.6–64.0] frames per second for the hands-free images and 47.9 [44.8–51.4] frames per second for the hands-on images.

**Table 1:** Patient characteristics

Variables	Values (*N* = 50)
Age (years)	67 (8)
Male/female (n, %)	38/12 (76/24)
Height (cm)	175 (9)
Weight (kg)	83 (16)
BMI (kg/m^2^)	27 (5)
Coronary artery disease (n, %)	31 (62)
Left main stenosis	10 (20)
1-vessel disease	5 (10)
2-vessel disease	8 (16)
3-vessel disease	18 (36)
Recent myocardial infarction (n, %)	15 (30)
Preoperative troponin-T (ng/L)	17 [11–61]
Preoperative NT-pro BNP (ng/L)	490 [1410–1387]
Preoperative LVEF (n, %)	
> 50%	35 (70)
31–50%	11 (22)
21–30%	4 (8)
Operation (n, %)	
Isolated CABG	24 (48)
Aortic valve replacement	20 (40)
Mitral valve replacement	4 (8)
Mitral valve repair	1 (2)
Surgery on the thoracic aorta	6 (12)
Redo cardiac surgery	3 (6)
EuroScoreII (%)	2.6 (2.7)
Aortic cross-clamp time (min)	70 (32)
Cardiopulmonary bypass time (min)	97 (45)
Propofol (mg·kg^−1^·hr^−1^)	2.4 [2.0–2.9]
Norepinephrine (mcg·kg^−1^·min^−1^)	0.03 [0.02–0.07]
MAPSE, pre-CPB (mm)	
Anterior	10.2 (2.6)
Inferior	11.7 (3.3)
Lateral	13.3 (3.5)
Septal	9.1 (2.5)
MAPSE, ICU (mm)	
Anterior	6.8 (1.7)
Inferior	7.5 (2.6)
Lateral	9.3 (3.0)
Septal	6.7 (2.1)

Values are mean (SD), n (%), or median [interquartile range]

*BMI* body mass index, *CABG* coronary artery bypass grafting, *CPB* cardiopulmonary bypass, *ICU* intensive care unit, *LVEF* left ventricular ejection fraction, *MAPSE* mitral annular plane systolic excursion measured manually on hands-on images, *NT-pro BNP* N-terminal pro B-type natriuretic peptide

### Monitoring feasibility

**Fig. 4:**
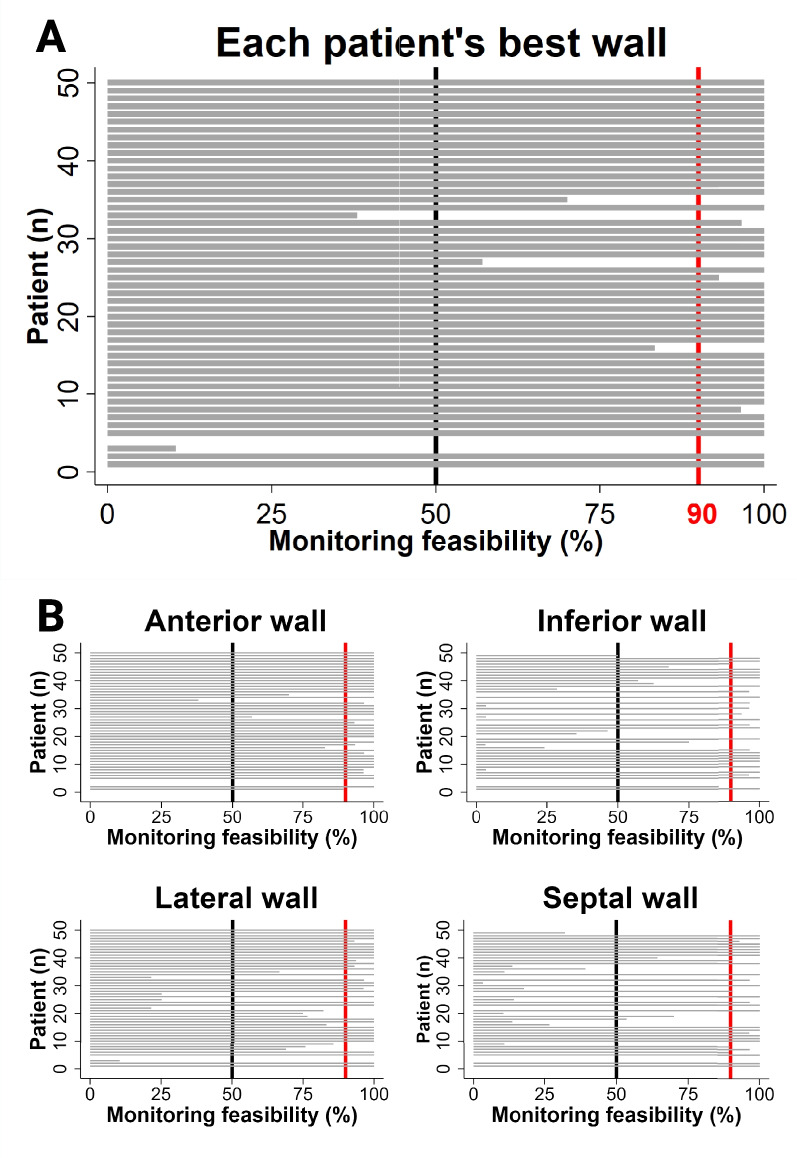
Horizontal bar graph of the monitoring feasibility in 50 patients. Each gray bar represents one patient (n). The length of the gray bars indicates the monitoring feasibility, i.e., the proportion (%) of the total recordings where mitral annular plane systolic excursion (MAPSE) could be reassessed from the same wall. The vertical black line indicates the threshold for acceptable monitoring feasibility (50%); the vertical red lines indicate the threshold for excellent monitoring feasibility (90%) **A** Monitoring feasibility of each patient’s best wall. **B** Monitoring feasibility of each wall within each patient

AutoMAPSE had excellent monitoring feasibility in 88% of patients, acceptable in 6%, and poor in 6% (Fig. 4A). Of the four walls, the anterior wall was the most feasible (Fig. 4B). Unscheduled probe adjustment due to probe dislocation was necessary in 14 of the 1230 sets of hands-free images (1.1%). The scheduled probe adjustments did not affect autoMAPSE measurements, as there was no difference between hands-free and hands-on autoMAPSE (0.2 mm, *P-*value 0.09, 95% CI − 0.03 to 0.4 mm). Due to the imaging windows, almost half of the patients (48%) had suboptimal image quality in the hands-free images; none of these patients were excluded.

### Bias and limits of agreement

**Fig. 5:**
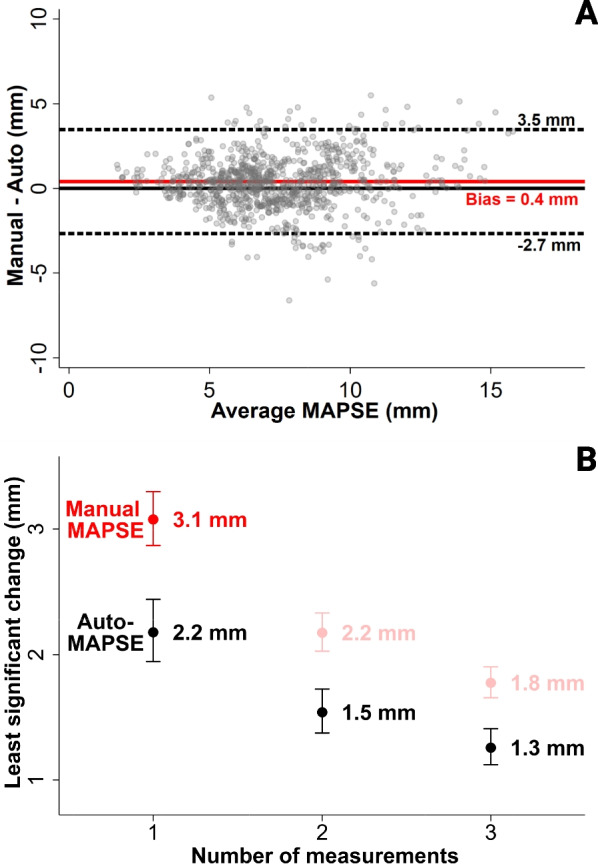
Comparing automatic measurements with manual measurements of mitral annular plane systolic excursion (MAPSE) in 50 patients. Data are pooled from measurements on hands-on and hands-free images. **A** Bland–Altman plot of 943 paired MAPSE measurements from the triplicate images. Each measurement is the average MAPSE of up to ten heartbeats. Red line, bias; dashed lines, limits of agreement that are adjusted for within-patient dependency. **B** Precision of each method reported as the least significant change. Lower least significant change means better precision. Each measurement is the average MAPSE of up to ten heartbeats. Brackets show the 95% confidence interval. *AutoMAPSE* automatic measurement of MAPSE

AutoMAPSE had a low bias (0.4 mm) and acceptable agreement with manual measurements (LOA − 2.7 to 3.5 mm) when pooling the hands-on and hands-free images for analysis. Visual inspection of the Bland–Altman plot showed that the differences were reasonably constant across the range of MAPSE (Fig. 5A). This comparison reflects the pooled agreement between autoMAPSE and manual measurements for all four measurement methods.

To compare hands-free monitoring with current echocardiographic practice, we compared hands-free autoMAPSE with hands-on manual MAPSE on the triplicate images. This analysis also showed an acceptable agreement between hands-free autoMAPSE and hands-on manual MAPSE (bias − 0.6 mm, LOA − 3.9 to 2.7 mm), supporting that hands-free image acquisition and measurements obtained similar values as the fully manual method.

### Precision

AutoMAPSE had a lower LSC than manual measurements, indicating that autoMAPSE was more precise (Fig. 5B,  P < 0.001). A subgroup analysis of the four methods revealed that the LSC (for one measurement averaging 10 heartbeats) was 1.8 mm for hands-on autoMAPSE, 2.4 mm for hands-free autoMAPSE, 2.9 mm for hands-on manual MAPSE, and 2.7 mm for hands-free manual MAPSE.

### Trending ability

**Fig. 6:**
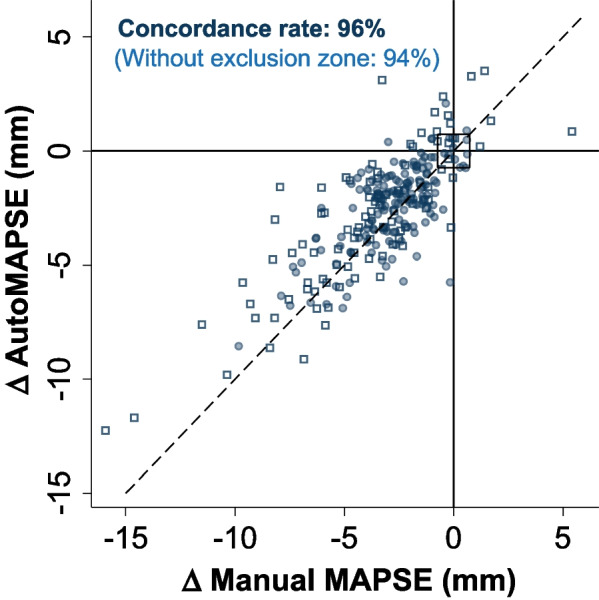
Trending ability between automatic and manual measurements of mitral annular plane systolic excursion (MAPSE). Four-quadrant plot showing 244 paired measurements from 50 patients, pooled from hands-on and hands-free images. The origo box indicates a central exclusion zone of 10% change. Each data point is the average measurement from one wall. Filled circles, hands-free images; open squares, hands-on images; dashed line, identity line; *AutoMAPSE* automatic measurement of MAPSE

AutoMAPSE had excellent trending ability for tracking changes in manual MAPSE (Fig. 6). AutoMAPSE calculated the change in MAPSE from at least one wall in a large proportion of patients (88% for hands-free autoMAPSE, 90% for hands-on autoMAPSE). Trending ability was similar for the subgroups of hands-free autoMAPSE (concordance rate 97%) and hands-on autoMAPSE (concordance rate 95%).

A decrease in MAPSE was detected using all four methods. When initiating CPB, the decrease in MAPSE was 2.4 mm using hands-free autoMAPSE and 2.7 mm using hands-free manual MAPSE (*P* < 0.001 for both). Similarly, between pre-CPB and the ICU, the decrease in MAPSE was 2.6 mm using hands-on autoMAPSE and 3.7 mm using hands-on manual MAPSE (*P* < 0.001 for both).

## Discussion

In this study, we found that continuous monitoring of LV function at the bedside was highly feasible using autoMAPSE. Compared with manual measurements, autoMAPSE had excellent trending ability, low bias, acceptable agreement, and was more precise. These findings suggest that autoMAPSE can potentially improve the monitoring of LV function in postoperative intensive care patients.

An essential finding regarding continuous monitoring was that autoMAPSE could automatically analyze images obtained in a hands-free manner. This finding allows autoMAPSE to obtain very frequent measurements of LV function. Frequent and automatic measurements are mandatory to detect unpredictable changes and thereby achieve the goal of monitoring. While automatic measurement of LV function by echocardiography has been explored previously [15–18, 33], the necessity for manual image acquisition has always limited the frequency of measurements necessary for continuous monitoring. By allowing frequent measurements through hands-free imaging, autoMAPSE addresses a longstanding limitation that has hitherto disqualified echocardiography as an efficient tool for continuous monitoring.

The precision of autoMAPSE is another factor mandatory for monitoring changes in LV function [31]. Echocardiography currently lacks the necessary precision to detect subtle changes in LV function due to significant variability in operators, manual measurements, and image acquisition. Relying on an imprecise method to distinguish actual changes in LV function from measurement variability requires large changes, and large changes typically occur late. AutoMAPSE enhances precision by minimizing operator variability, averaging multiple automatic measurements, and ensuring consistent image acquisition. Consequently, smaller changes in LV function may be detected earlier, which could facilitate earlier and more effective therapy.

### Generalizability

Several aspects indicate that our findings may be generalizable. First, the patients in this study differ significantly from the patients in the training data [10]. Yet, autoMAPSE has demonstrated high feasibility, not only in this present study but also in cardiology patients [10] and in patients after isolated coronary bypass grafting [11]. Thus, autoMAPSE is likely generalizable to other critically ill patients. Second, image quality was suboptimal in many of our patients (48%), probably because prolonged scanning time impairs the mucosal contact. Thus, suboptimal image quality is likely inherent to continuous TEE over time. Yet, despite suboptimal image quality, autoMAPSE demonstrated excellent monitoring feasibility in 88% of the patients. Finally, images for autoMAPSE are easy to obtain, and clinicians can achieve acceptable proficiency in TEE after only a few hours of training [34]. Thus, autoMAPSE may allow non-expert clinicians to quantify LV function with high feasibility and precision.

Previous studies support the safety and feasibility of continuous TEE using an indwelling, miniature TEE probe [34–36]. These studies, however, have some limitations that autoMAPSE could potentially improve. First, their assessments were still intermittent because their images required manual acquisition [34, 36]. In contrast, hands-free autoMAPSE overcomes this limitation and allows effortless image acquisition for continuous monitoring. Second, in the previous studies, the assessments of LV function were either qualitative [35] or semi-quantitative [34, 36]. Whenever continuous variables are categorized, information is lost, and precision is reduced. Thus, the previous studies may have overlooked small, yet important, changes in LV function. AutoMAPSE offers an improvement by obtaining precise, quantitative measurements of LV function. Combining autoMAPSE with these smaller probes could improve safety even further.

### Clinical implications

AutoMAPSE will presumably be most useful in situations when the patient is immobilized, such as during surgery or the resuscitation phase of critical illness. When the clinician first inserts the probe, he or she can perform a hands-on TEE exam. At this stage, LV function can be precisely quantified using autoMAPSE and the quality of the automatic measurements can be evaluated by the clinician by inspecting the detected annulus points. After the hands-on exam, the probe can be left in situ for continuous monitoring of MAPSE. If MAPSE subsequently worsens, the clinician can integrate MAPSE with other clinical and echocardiographic information to identify the likely reason for deterioration. Likewise, if therapies are adjusted, their effects on LV function can be monitored effortlessly using either hands-on or hands-free autoMAPSE. Thus, autoMAPSE can be combined with current practices to improve cardiovascular management.

### Limitations

Our study has several limitations. First, although none of our patients had complications from TEE, complications may occur. Fortunately, these are usually self-limiting, and severe bleeding or esophageal perforation is very rare [35–38]. Of note, complications are mainly related to probe insertion, flexion, and manipulation [21, 22]. Therefore, by leaving the probe tip unlocked and focusing on hands-free imaging, we expect the risk of serious complications to be minimized. Continuous TEE over time may also be safer using smaller probes. Second, although manual measurements are accepted as the reference method when validating AI in echocardiography, manual measurements are not the gold standard. The imprecision of our manual measurements widens the LOA, independent of autoMAPSE [29, 30]. However, this imprecision does not affect the bias or the constancy of the differences in a Bland–Altman plot [27, 29]. The effects of this limitation are evident in our results (Fig. 4A and B). Third, MAPSE as a parameter of LV function assumes a stationary LV apex and that the maximal amplitude of the mitral annulus reflects end-systole and end-diastole. These assumptions are usually satisfied, but autoMAPSE may quantify LV function somewhat inaccurately in patients with post-systolic shortening, pre-systolic stretching, a non-stationary LV apex, or intraventricular dyssynchrony. Foreshortening of the LV is another source of inaccuracy, but this is minimized by using the 2C. However, the impact of these inaccuracies has not been well defined, especially when autoMAPSE is used for continuous monitoring. Fourth, the scheduled probe adjustments is a source of bias that increases the monitoring feasibility observed in our study. On the other hand, the value of autoMAPSE is highest in critical situations where LV function changes rapidly. In these situations, the attending clinician tends to be bedside present. Therefore, even with small probe adjustments, monitoring LV function would still be effortless compared to current options. Regarding this, we found that such probe adjustments had minimal impact on the measurements.

## Conclusion

Continuous monitoring of LV function was feasible using hands-free autoMAPSE and it was most feasible for the anterior wall. Compared with manual measurements, autoMAPSE had excellent trending ability, low bias, acceptable agreement, and was more precise.

## Take-home message

Echocardiography has limited capabilities for continuous monitoring of left ventricular function in intensive care patients. To resolve this issue, we present a new method that combines transesophageal echocardiography with artificial intelligence to automatically measure mitral annular plane systolic excursion, called autoMAPSE. In this present study, we found that autoMAPSE could provide continuous monitoring of LV function in intensive care patients.

### Supplementary Information

Supplementary Material 1. A video demonstrating hands-free autoMAPSE in real-time. The transesophageal echocardiography probe is stabilized using a custom-made probe holder. Hands-free images are streamed into a research laptop. Automatic measurements of MAPSE are thus acquired at the bedside. The patient has provided consent for publication.

## Data Availability

The datasets used and analyzed during the current study are available from the corresponding author upon reasonable request.

## Abbreviations

2C: Midesophageal two-chamber view
4C: Midesophageal four-chamber view
AI: Artificial intelligence
AutoMAPSE: Automatic measurement of mitral annular plane systolic excursion
CPB: Cardiopulmonary bypass
ICU: Intensive care unit
LOA: Limits of agreement
LSC: Least significant change
LV: Left ventricular/Left ventricle
MAPSE: Mitral annular plane systolic excursion
SD: Standard deviation
TEE: Transesophageal echocardiography
